# Child marriage of female Syrian refugees in Jordan and Lebanon: a literature review

**DOI:** 10.1080/16549716.2019.1585709

**Published:** 2019-03-25

**Authors:** R. El Arab, M. Sagbakken

**Affiliations:** a Al-Ghad International Colleges for Health Sciences, Riyadh, Saudi Arabia; b Department of Nursing and Health Promotion, Faculty of Health Sciences, OsloMet – Oslo Metropolitan University, Norway

**Keywords:** Child marriage, Syrian refugees, gender, reproductive health, reproductive rights

## Abstract

**Background**: The Syrian conflict has resulted in major humanitarian crises. The risk is particularly high amongst female children who face additional gendered risks, such as harassment and sexual violence, including a rise in prevalence of child marriage. Despite the importance of this topic, current literature remains relatively scarce.

**Objectives**: This study aims to explore the social and healthcare repercussions of Syrian refugee child marriages in Jordan and Lebanon.

**Methods**: A systematic review of the literature was carried out to gather evidence, from a total of eight articles. Data analysis was conducted using the Critical Appraisal Skills Programme check tool to systematically assess the trustworthiness, relevance and results of the included papers.

**Results**: The findings of this research identify tradition, honour, economics, fear, and protection-related factors as drivers of child marriage of refugees in Jordan and Lebanon. These motives overlap with findings regarding access to reproductive health and reproductive rights. The lack of autonomy of the child to give informed consent is augmented in the context of protracted violence and displacement.

**Conclusion**: There is a need for a holistic approach to provide safe spaces, education, and protection to young girls and their families to reduce their acceptance of child marriage.

## Background

Findings show that the number of Syrian refugees fleeing to other countries continues to rise [1]. As of July 2018, there were 668,123 registered Syrian refugees in Jordan and 976,000 refugees in Lebanon [2,3], indicating that the highest density of the Syrian refugee population is in these countries. A common challenge in conflict regions is the rise in the presence of child marriage rates, for various reasons [4]. Svanemyr et al. [5] conclude that even though child marriage is not widespread in the contemporary Arab region, there has been a rise in its presence in recent years due to the nature of conflicts and the need for protection. Prior to the crisis in Syria, child marriage took place amongst 13% of girls under the age of 18, but since then, forced displacement has led to a major increase in this number [6]. The United Nations Fund for Population Activities (UNFPA) now reports that 35% of Syrian refugee girls are married before the age of 18 [7].

Lack of official marriage registration makes it difficult to obtain valid information about the prevalence of child marriage, as many do not have the documentation required to complete some of the initial registration steps. For instance, in Lebanon, Syrian refugees face a number of challenges when registering births and marriages, limiting their access to services such as shelter and education [6]. Many Syrian refugees do not have valid residency and many parents do not have valid proof of marriage, which is required to complete some of the initial birth registration steps such as obtaining a birth certificate [6].

This potential rise in child marriages may be linked to the fragility characterising these refugees’ life situation. Marrying a girl at a young age may be perceived as increasing the security of the girl and/or her family [8]. A qualitative study exploring child marriage practices found that protection, security and financial hardships were the most common motives for child marriage [7]. In their assessment of female refugees from Syria, UNICEF found that many girls view early marriage as an opportunity to escape poverty and workforce exclusion [9].

According to DeJong et al. [10], various approaches to defining child marriage have been adopted. While most studies define child marriage as a marriage in which one or both parties are married before the age of 18 [11], others contend that informed consent should be a part of the marriage union, and that this is lacking in child marriage. UNICEF [12] concludes child marriages are those where one or both parties are underage and have not personally expressed full, free and informed consent to the union. This review study adopts this holistic definition, where a focus on age is not the only factor, but consent of the individual is also taken into account.

Child marriage can be linked to a range of poor social and health outcomes for women and girls, including high risks of early pregnancy [13], mental health issues due to forced early sexual debut [14], and maternal death and disability, including post-pregnancy complications [15]. Additional sexual health challenges, such as the lack of agency of brides to negotiate the use of condoms even in conditions of high risk of HIV infection, are reported [16]. Such challenges are associated with the potential presence of increasing risk of domestic violence and power differentials in the relationship, which could be attributed to differences in age. There is evidence which shows that early marriage is linked to negative social outcomes, including school dropout, lack of employment and low educational attainment [15,16].

Prior evidence has shown that while child marriage during conflict and displacement is not unique [11], the nature of outcomes and the reasons for such practices are found to be varied based on contextual differences [17]. The rise in child marriage amongst Syrian refugees has been studied previously for a range of reasons, including understanding underlying motives and determining the extent and prevalence of such forced marriages [18,19]. Given potential variations in the implications of child marriage by region, it is essential that specific objectives on outcomes and antecedents of child marriage are identified.

The primary aim of this review is to present a holistic analysis of the reasons behind child marriage amongst Syrian refugees. Secondarily, this review aims to explore the refugee populations’ access to autonomous decision-making regarding health and family planning in Lebanon and Jordan. Thus, the major focus of this review is to determine the motives which support female child marriage among Syrian refugees in Jordan and Lebanon and to assess reproductive decision-making, including access to contraceptive use, by Syrian child bride refugees in Jordan and Lebanon.

## Methods

As Clark and Oxman [20] argue, the search boundaries for an investigation need to be set to conduct a comprehensive systematic review of literature. The review process used here, which included an ongoing discussion between the two authors, adopted the PICO model (patient/problem, intervention/exposure, comparison and outcome; see Table 1) to evaluate the research objectives [21]. The use of the PICO model helped streamline the search process to maximise the likelihood of finding relevant articles.

**Table 1.** PICO framework and search terms.

Objective	Patient/problem	Intervention/exposure	Comparison	Outcome
1	Refugees in Jordan and Lebanon	Child marriage OR Early marriage OR Child bride	No comparator	Causes OR Reasons OR Motives
2	Refugees in Jordan and Lebanon	Child marriage OR Early marriage OR Child bride	No comparator	Reproductive health OR Reproductive rights OR Contraceptive access

This study adopted a systematic literature review methodology. The search terminologies identified in Table 1 were used to search different journals and online databases, including PubMed, ScienceDirect, CINAHL and EBSCO. Raj et al. [22] contend that there is a gap in health-related outcome reporting amongst refugee communities; Therefore, this study adopted a grey literature approach to identify additional articles. Grey literature and reports published by international non-governmental organisations (NGOs) were included, with a focus on Save the Children (STC), the United Nations and other relief organisations. Once the search terminology and search database were established, the inclusion and exclusion criteria were determined. The key inclusion and exclusion criteria are detailed below.

### Inclusion criteria

- Studies discussing child marriage amongst refugees in Jordan and Lebanon.
- Clear description of reasons/motives or reproductive rights/decisions.
- Reference to challenges of refugees.
- Peer-reviewed or reports from International NGOs.

### Exclusion criteria

- Previously published meta-analyses or systematic reviews on the same subject.
- Non-English articles.
- Research published before 2012.

Fink [23] contends that the basic guideline for approval can be determined using the method of quality evaluation. Quality assessment requires following the right approach to identify corroborative evidence. According to Cochrane [24], the method of quality estimation should cluster and categorise articles on the basis of quality and arrangement. This research adopts the Critical Appraisal Skills Programme (CASP) guidelines to address the quality of the studies chosen to address methodological rigour assessment [25]. Use of the CASP tool can help provide a complex assessment of study evidence which can contribute to acceptance or rejection of the studies under question. Table 2 presents the findings of the CASP analysis. Table 2

**Table 2.** Critical Appraisal Skills Programme  (CASP), Save The Children (STC) Focus group discussion (FGD).

Criteria	Mourtada et al. [6]	STC [28]	Bartels et al. [31]	Acousta and Thomas [29]	Cherri et al. [27]	El-Mowafi et al. [26]	Sahbani et al. [30]	UN Women Report [32]
Statement of aim of research	The aim of the study is to gain information about the factors that promote child marriage practices amongst Syrian refugees in Lebanon.	The aim of this report is to address the growing problem of child marriage among Syrian girl refugees in Jordan.	This study explores the underlying factors contributing to child marriage among Syrian refugees in Lebanon with the goal of informing community-based strategies to address the issue.	The aim of the research was to present a comprehensive assessment of the causes and consequences of early child marriage in Syrian refugee communities in Jordan.	To determine the sexual and reproductive health needs of Syrian refugees in Lebanon (aged 15–49).	To explore the Syrian child bride knowledge and attitude towards experiences of contraceptive use by examining refugee camps in Jordan.	To understand the challenges faced by international humanitarian agencies. The challenges to be addressed include the health issues faced by child marriage refugees in Jordan.	The aim of the study was to provide an assessment of child protection issues in Jordan and identify the needs of child brides in such conflict regions.
Appropriateness of methodology	Qualitative design is ideal as it helps provide insight into the views of various stakeholders (child brides, families) and service providers).	Narrative research. The goal is to provide a comprehensive overview of the existing situation in Jordan.	Survey research. The researcher developed a mixed-methods data tool (collecting both qualitative and quantitative data).	Narrative review. Early marriage causes and consequences were detailed.	Qualitative research through FGD can provide information on the challenges faced by child brides and provide opportunities for more in-depth discussion.	Qualitative research using focus group discussion.	Narrative review.	Survey research using a questionnaire, and qualitative data were obtained through in-depth interviews with key community
Research design was appropriate to address the research aim	Yes. The design is well organised.	Unclear. The criteria behind the themes chosen for discussion are limited.	Yes. The design is appropriate as it attempts to understand views of participants while identifying trends, requiring both qualitative and quantitative data.	Unclear. The criteria behind the themes chosen for discussion are limited.	Yes. The research details the need for qualitative over quantitative research.	Unclear. The details on research design and rationale are not provided.	Unclear. The criteria behind the themes chosen for discussion are limited.	Choice of questionnaire was not explained. However, the appropriateness of the design and the need for survey-based methods to increase generalisation of findings were addressed.
Population recruitment strategy was appropriate for the research	Moderate. The selection of focus group participants and the type of sampling is not explicit.	N/A	Choice of families and child brides is through convenience sampling means. The rationale behind the same is less discussed.	N/A	Yes. The number of participants approached, who participated and who withdrew is clear. The rationale for the choice of these participants is also clear.	Not provided.	N/A	Clarity in choice of instruments and their purpose is evident.
Data collection was done in a way that addressed the research issue	Yes. Clarity in data collection is evident.	Unclear. No clear selection of search words or inclusion criteria. Given that the goal of the report was to act as a white paper and provide information, appropriate information is available.	Yes.	The choice of material and the actual search process is unclear.	Yes. The data collection was carried out using an interview template developed and tested beforehand.	Yes. The researcher argues that the focus group guide was developed based on evidence from prior research evidence on the subject.	Unclear. No clear selection of search words or inclusion criteria. Given that the goal of the report was to act as a white paper and provide information, appropriate information is available.	Yes.
Ethical concerns were considered	Yes. The review board was identified. Informed consent and confidentiality were assured.	N/A	Yes. Queen’s University Health Sciences and Affiliated Teaching Hospitals Research Ethics Board were identified as the key ethics board. Consent was obtained in Arabic.	N/A	Yes. Verbal consent was gained. Assurances regarding confidentiality and anonymity were made.	Not available.	N/A	Ethical concerns were well addressed. Informed consent and confidentiality were assured.
Data analysis was sufficient and rigorous	Yes. Thematic analysis was carried out.	Thematic analysis.	The data were analysed using quantitative and qualitative methods to provide trend analysis as well as thematic analysis.	Thematic analysis.	Yes. Inductive, open-theme analysis was carried out.	Thematic analysis.	Thematic analysis.	Descriptive analysis, thematic analysis.
Clear statement of findings	Yes.	Yes.	Yes.	Yes.	Yes.	Yes.	Yes.	Yes.
Value of research	Recommendations are provided on how child marriages can be avoided in refugee communities.	Recommends actions by various stakeholder groups to address inherent motives regarding child marriages.	A range of factors contribute to child marriage, including poverty, lack of educational opportunities and concerns about violence.	The occurrence of child marriage is linked to a lack of financial support and guidance, while proper access to security is also identified as a theme. The report addresses the need for better understanding of the financial and emotional wellbeing of communities.	Interventions to improve health and reproductive rights of child brides were essential in refugee communities, including awareness and access.	There is a need to address both emotional needs and knowledge gain. Attitude and practice-related interventions are essential.	Identify health agency-level and humanitarian actor-level actions that can be taken.	Overarching recommendations are detailed on the challenges of child marriage and its impact in terms of physical and sexual violence.
Overall implications	Methodological rigour could have been improved by using better measures of sampling.	Need for clarity in choice of literature.	Methodologically sound. However, the research instrument was piloted with a small sample.	Need for clarity in choice of literature.	Methodologically sound. The rationale behind the research method and the presentation of findings were easy to understand.	Need for clarity in participant choice and data collection.	Need for clarity in choice of literature.	Methodological rigour is high.

**Figure 1.**
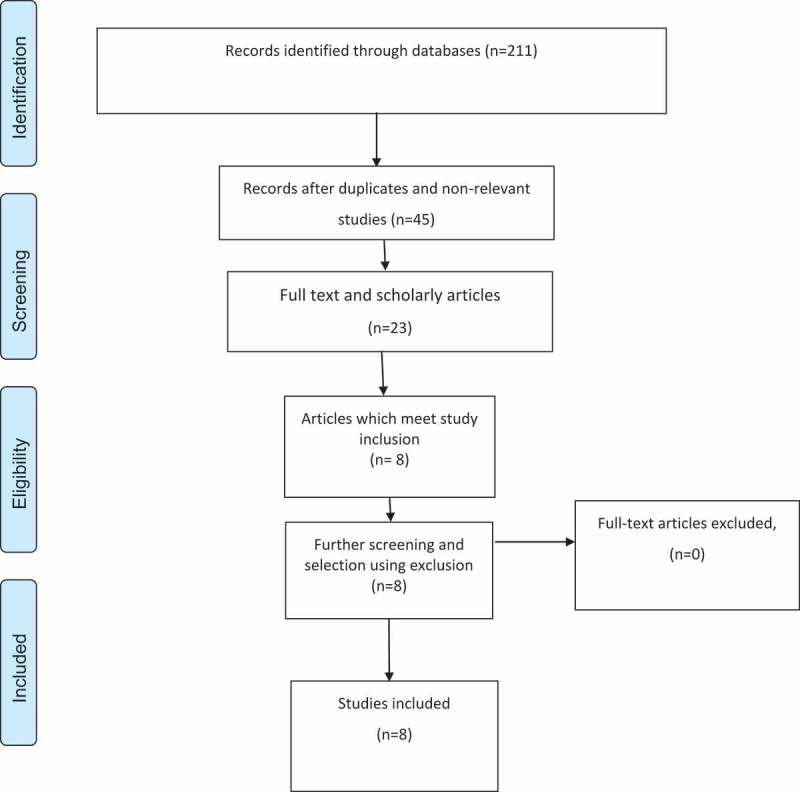
PRISMA flowchart.

From the above CASP analysis, it is evident that while most articles have some challenges with their methodological rigour, they provide clarity in aim, choice of method, analysis and findings. It was concluded that despite the methodological challenges, these articles should be chosen for the review given their ability to provide insights and evidence on the proposed research objectives. Figure 1 presents an assessment of the PRISMA framework.

## Results

A thematic approach was used to identify key themes. Each study was examined for relevant themes that could answer the research objectives. The following Table 1 summarises the findings with a summary of the themes. The total number of articles included in this review was eight. Most of the studies examined were qualitative, cross-sectional [6,26,27] or narrative reviews [28–30]. There were two studies that adopted a mixed-methods approach, collecting both qualitative and quantitative data [31,32]. Of the eight studies discussed, five related to the motives behind child marriage [6,28,29,31,32]. Three studies focused predominantly on reproductive rights-related challenges including access to contraceptives and their use [26,27,30]. Three studies were based in Lebanon [6,27,31] and five in Jordan [26,28–30,32]. The key themes identified with respect to motivation for child marriage ranged from protection (physical or economic) to tradition and protection of honour.

### Motives for child marriage amongst Syrian refugee communities in Jordan and Lebanon

#### Physical protection

The lack of male support was reported by many self-identified child brides as a potential reason for child marriage. Bartels et al. [31], who focused on Syrian girls in Lebanon, found that physical harassment in camps, as well as sexual advances made by men and guards against younger children, are some of the key reasons why parents pressure their children to get married. Mourtada et al. [6] concluded that child brides from Syria often face greater challenges with respect to sexual harassment, as they are rarely given the opportunity to make formal complaints. Gaining a male protector through marriage is often considered an ideal approach to overcoming potential attacks from unknown individuals. STC [28] indicate that given the rise in the number of casualties of the ongoing war, there are many families with a limited number of men in the household. The perception of such men being unable to provide sufficient physical protection for women and girls in their family encourages the acceptance of early marriage as a trend. In a United Nations (UN) Women Report [32], the findings on reasons for early marriage among men and women showed that marriage is a form of protection for girls (28.7%) in the community. Therefore, the perception that child marriage can enhance the physical protection of the child seem to be a key motive which has led to the increase in child marriage.

#### Community expectations

Community expectations and worry associated with honour are another common motive highlighted across the chosen studies. In their study, Mourtada et al. [6] reported that interview participants acknowledged their unwillingness to support child marriage but were often forced to do so by community trends. The interview participants highlighted the concept of *al Sutrah*, which indicates a desire to protect the honour of their women. Adolescent women’s reputations are a cornerstone of familial reputation, and a rise in physical harassment has led to community-level adaptations to child marriage. Thus, many families look to child marriage to reduce the risk of physical harm to their female children through early marriage. Bartels et al. [31] contend that many men consider child marriage in Lebanon to be a way of protecting children from rape and the threat of sexual violence. Girls exposed to different forms of sexual abuse and harassment may find it difficult to marry later due to societal shunning, resulting in the rise in acceptance of the practice [31].

The STC [28] report supports this notion and concludes that familial honour protection is a key driver in supporting acceptance of child marriage at the societal level amongst Syrian refugees in Jordan. Based on a quantitative survey, the UN Women Report [32] also concluded that the most common reason for early marriage given by women and men was to protect family honour (women: 39.3%) and as a part of custom and tradition (women: 44.2%; men: 33.4%).

Acosta and Thomas [29] reported that many Syrian refugees in countries like Lebanon and Jordan are raped and then forced to marry. Bartels et al. [31] also contend that single women are forced to marry off their daughters due to rape and/or threats. Associated challenges of being shunned by society may hasten parents’ decision to allow their daughters to marry sexual offenders [31].

#### Financial needs/economic reasons

Economic challenges and hardship are often listed as key motives influencing decisions on forced early marriage. Bartels et al. [31] concluded that the perceived lack of protection often relates to financial access, as young girls have limited resources to support themselves. Since these girls are forced to move to Lebanon due to the conflict, they have limited schooling and educational opportunities, providing them with only low-skilled employment options [31]. If they receive marriage propositions from well-settled men, they may choose this option out of desperation [31]. Mourtada et al. [6] also concluded that conflict and displacement result in major challenges to refugee family operations, and participants in focus group discussions reported dire living conditions, poverty and insecurity, which act as potential motivators. Given that household finances are dire and likely to become worse, marrying off a daughter at an early age can help reduce the overall financial burden on the family.

Acosta and Thomas [29], in their assessment of the refugee communities, concluded that Syrian families in Jordan and Lebanon face severe economic distress. Since most of them depend on family savings, and given that work is hard to find, it may be difficult to support the entire family. In this context, mothers believe that marrying off children is essential as it improves the child bride’s access to better economic opportunities. The UN Women Report [32] indicates that economic reasons for marrying at a young age include access to housing for women (20.1%) and providing options to alleviate financial problems (28.4%).

#### Parental support and family decisions

While many studies discussed families’ individual decisions to support child marriage for the purposes of poverty alleviation or overall family support, some studies identified pressure on young girls to fulfil family expectations and ease their overall decision-making. For instance, Mourtada et al. [6] concluded that since there was rising fear of potentially unfavourable gossip from local communities due to rumours of social misconduct, many women chose to get married to reduce the overall burden on their family. The STC [28] report also found that young girls recognise the lack of opportunities for education and employment and are willing to get married at an early age to reduce poverty challenges faced by their family. El-Mowafi et al. [26] concluded that there are often limited choices available to young brides as families abdicate responsibility, which leads to their decision to accept marriage.

### Reproductive rights challenges

The second key objective of this research was to assess health outcomes with specific reference to a child bride’s contraceptive needs as well as her knowledge about, access to, and use of contraceptives.

#### Lack of acceptance

Cherri et al. [27] and Sahbani et al. [30] found that there is limited acceptance regarding the use of contraceptives. The authors argue that many girls have to obtain permission from their husbands, which may not be given. El-Mowafi et al. [26] concluded that requesting access to contraceptive use can create additional challenges of physical violence. Sahbani et al. [30] argue that the husband’s refusal prevents married girls from using contraception and, given their lack of agency, child brides are forced to comply with their husband’s wishes. Amongst married women with no children, the use of birth control can be difficult, as they are under societal pressure or religious pressure not to use it [30].

#### Knowledge of contraception is low

According to Sahbani et al. [30], there are differences in knowledge about and attitudes towards contraceptive use. While there is a certain level of acceptance that contraceptive use can prevent unforeseen health consequences, including sexually transmitted diseases, this knowledge is low. However, there is some awareness of its use and its impact on pregnancy prevention. Sahbani et al. [30] also concluded that young girls are rarely given a choice about getting married and that the choice of contraceptive use is also limited. The authors further suggest that challenges linked to a lack of access to education on healthcare options and the constant pressure to get pregnant due to societal norms further reduce the option of transmitting knowledge and information about contraceptives and their use [30].

#### Access to contraception is low

Despite varying levels of knowledge on modes of contraceptive use, the most important barrier is the lack of access to such contraceptives. Sahbani et al. [30] concluded that on top of the pressure to marry, many girls are forced to procreate and have children, which is a primary objective of the husband in their family. Given this acceptance at the community level, most girls are unaware of international aid-driven efforts to increase access to contraceptive use. Cherri et al. [27] contend that even if there is access to contraceptives, there are challenges of cost. These authors concluded that low-quality intrauterine devices (IUDs) were not effective, and those which were of good quality were highly priced. Many women were unaware that IUDs were given for free in primary healthcare centres.

## Discussion

The findings of this review show that there are several motives which drive child marriage decisions; some are unique to the Middle Eastern context, including family honour, societal norms and supporting family decisions, and some are linked to financial challenges and burden, physical assault and safety-related challenges. Some of the challenges regarding reproductive rights relate to the lack of acceptance of, knowledge about, and access to contraceptives. A common theme that most of the studies in this systematic review identify is that there is a potential change in marriage practices amongst refugee communities [6,28,31,32]. Prior findings at a global level have shown similar motives, including concerns over safety, worsening economic conditions and a lack of future options for education or employment [11].

Bedri et al. [33] conclude that many families in Sudan believe in the notion of *al Sutrah*, or the social protection and preservation of the honour of the family through the honour of the bride. Evidence from refugee camps has shown a rise in concern about the safety of women; furthermore, there is increasing evidence of rape being used as a tool to control and terrorise families [34]. Traditionally, most Arab societies consider the virginity of the bride a key element which symbolises her purity [35]. To address the challenge of increasing insecurity and vulnerability, many families resort to early marriage to protect their daughters and promote their family honour. Family expectations and societal norms are also key themes which overlap with the lack of reproductive rights and agency of child brides. Evidence presented in this systematic review [26,27] has shown that women who express a desire to delay pregnancy may face the challenge of being abandoned by their husband. In many emergency contexts, families believe that they are protecting the girl’s honour when she is forced to marry or stay married to an individual. As Sahbani et al. [30] rightly argue, marriage cannot guarantee child safety, with some women being abandoned after giving birth to children. Cooperative for Assistance and Relief Everywhere (CARE) [11] argue that implementation agencies and donors who fight against child marriage should be willing to discuss the motives behind early child marriage and its potential implications for long-term child wellbeing, including access to reproductive rights and reproductive health.

Different studies [26,31] conclude that the physical safety and wellbeing of young girls is often a common element associated with the motives behind child marriage. Many studies reviewed in this research conclude that worry over physical safety and protection from harassment remain primary reasons behind supporting child marriage [26,31]. Zawati [34] further argues that in internal and transnational armed conflicts, rape continues to be an instrument of patriarchal domination and becomes a widespread weapon of terror and torture which rebels use to gain allegiance. The use of rape in refugee camps has been associated with intimidation and gaining favour. Interestingly, the family pressure and worry regarding physical threats is more evident than that experienced by the child brides themselves. Another key finding that should be discussed is the perception of protection. For example, Bartels et al. [31] argue that though family members (especially men) view child marriage as protection against sexual harassment, many women view it as an overreaction. They believed that young girls were being protected too much and that girls were often perceived to be not protected enough. This supports evidence that calls for a better balance between family expectations and those of the child. Aid agencies that work in the region should consider giving practical support that can provide immediate resolution to such problems.

The impact of lack of education is also an underlying factor which creates challenges in regards to child marriage and continues to have implications for access to reproductive rights. Mourtada et al. [6] conclude that there is a significant minimisation in the movement of women in Lebanese and Jordanian society, which reduces opportunities for education or employment. This makes them dependent on men, resulting in a lack of agency and the continued presence of patriarchal implications associated with child marriage. This lack of agency has been linked to transactional sex and short-term contractual marriages which leave child brides with children and in poverty. Bartels et al. [31] identify that there are girls who are married for brief periods of time to multiple men for money paid to their families. The girls have no autonomy in their decision-making in regards to any aspect of their life. Kidman [36] also concludes that such girls have limited access to contraceptive use and no knowledge about preventing pregnancy or potentially dangerous health challenges. Zuhur [37] further indicates that this can lead to additional challenges of inadequate childbirth spacing, transmission of infection and HIV/AIDS risks. Any intervention that is targeted towards young girls in the context of being a refugee should address ways to improve the rights of such children, to legalise the age of consent and create strict enforcement laws surrounding their implementation.

The lack of education and rising dependence in the form of financial needs leads to a limited understanding of informed consent. An assessment of child marriage amongst such refugees shows a lack of knowledge and ability to give informed consent [37]. A child bride is considered to be unable to consider the gravity and implications of sex and may be unable to consent to have sex [37]. Similarly, the lack of autonomy associated with the use of contraceptives and the associated challenges of pregnancy [38] can be attributed to the limited opportunity for consent. Many girls who agree to get married are misinformed about their choices [38]. The suggestion by the community and the family that girls who are victims of protracted violence will be safer and their honour will be intact further contributes to misinformed brides [38]. Such girls are often subjected to rising challenges of continued physical and emotional domestic violence, with a lack of empowerment regarding the use of contraceptives [37]. This evidence supports the need for safe spaces within refugee centres which can serve as appropriate units for the introduction of life skills training, vocational training and, more importantly, better education on sexual and reproductive health.

Kidman [36], in a global assessment of the challenges linked to programmatic interventions proposed to address child marriage amongst refugees, argues there is a need for context-specific assessment of child needs. For example, the findings provided by Bartels et al. [31] and Mourtada et al. [6] clearly highlight a mix of challenges, including honour-based issues and economic hardships. However, a UN [32] study which focused on Jordan found that the most common factor associated with child marriage was supporting tradition and customs. These findings suggest the need to move beyond a one-size fits all approach and to implement context-specific plans which are customised to the needs of the region and the refugee support centre.

Globally, the UN has designated an internal panel for gender and development to convene and identify ways through which child marriage could be eradicated [39]. However, the focus of the pilot project was on Afghanistan, Ethiopia, Ghana, Somalia and Zimbabwe [39]. There are some limitations associated with this study which should be acknowledged. This systematic review focused on five peer-reviewed and three grey literature databases and included a relatively limited number of studies. Thus, the knowledge gained might be incomplete and important perspectives may be absent. Further, this review did not compare child marriage motives among non-refugees in Jordan and Lebanon, in which could have provided a more comprehensive picture of the motives of the refugee population in particular. Based on this review we recommend a comprehensive assessment of child marriage motives by comparing Syrian refugees in Jordan and Lebanon with child marriage in other countries. This can help provide region-specific insight and help differentiate between country-specific challenges; tradition-specific challenges and specific challenges in the refugee population. Future research need to explore the differences of marriages carried out between children and those taking place between adult men and female children.

## Conclusion

Child marriage can be a gateway to other forms of gender-based violence and can have lifelong adverse impact on the child and her offspring. This review has identified some common reasons associated with child marriage and factors that reduce the autonomy of child brides with respect to their reproductive rights. The key themes identified with respect to drivers for child marriage ranged from physical and economical protection, to tradition and safeguarding of female honour; augmented by the social context of protracted suppression, lack of autonomy, violence and fear.

Key issues that inhibits reproductive rights relates to constraints in regards to a lack of access to education, contraception and health care, limited knowledge, and a lack of agency. These findings suggest that policy makers and refugee agencies should establish safe spaces for would-be child brides, supporting the girls and parents’ willingness to speak out about sensitive issues, providing better family planning and contraceptive education.

## Paper context

This study adopted a systematic literature review methodology to discuss the reasons/motives and reproductive rights/decisions associated with child marriage amongst refugees in Jordan and Lebanon.

## Appendix Appendix I. Study Summary

**Table:**

Authors	Aim	Research design	Study methodology	Findings	Themes discussed
Mourtada et al. [6]	The aim of the study is to gain information about the factors that promote child marriage practices amongst Syrian refugees in Lebanon. The study also attempts to identify recommendations on mitigating these drivers leading to child marriage practices.	Qualitative research and cross-sectional design.	Eight focus group studies were conducted with married and unmarried women, and mothers and fathers. Eleven key informant interviews were conducted with service providers.	Child marriage was a growing cause of concern amongst refugees in Lebanese camps. The key reasons cited include economic and safety-related factors.	Motives for child marriage: reduction in age of marriage, worsening of economic conditions, disrupted education, feelings of physical insecurity, and challenges related to community-linked decision making.
STC (Save the Child) [28]	The aim of this report is to address the growing problem of child marriage among Syrian girl refugees in Jordan.	Narrative review.	The research adopts a narrative review approach, where the goal is to highlight the underlying lack of opportunities that are available to these Syrian refugees.	The findings of the report revolve around identifying potential challenges which are systemic in contributing to the rise of child marriage, and the need to reduce their negative impact.	Motives for child marriage: parental pressure, economic and tradition-led decisions, physical protection.
Bartels et al. [31]	This study explores the underlying factors contributing to child marriage among Syrian refugees in Lebanon with the goal of informing community-based strategies to address the issue.	Survey research, cross-sectional.	The study used a mixed-methods data collection approach, where 1422 self-interpreted stories were collected from married and unmarried girls. These data were analysed using quantitative and qualitative methods to provide trend analysis and thematic analysis.	The findings from the quantitative analysis show that there are different perspectives associated with female and male participants’ interpretation of child marriage challenges. From the qualitative assessment of the independent stories, it is observed that there is a continued perception of overprotection, challenge of economic hardship and meeting societal concerns and accepted norms. Syrian girls and mothers were more likely to share honest experiences and their perspectives related to protection, security and harassment, as well as education. Male participants highlighted financial security challenges.	Motives identified: financial hardship, lack of educational opportunities, parental pressure, safety concerns around sexual and gender-based violence (SGBV), honour and tradition.
Acousta and Thomas [29]	The aim of the research was to present a comprehensive assessment of early child marriage causes and consequences in Syrian refugee communities in Jordan.	Narrative review of the literature.	The research adopts a narrative review approach, where the goal is to highlight the underlying challenges linked to child marriage faced by refugee child brides.	The issue of early/child marriage is recognised in the research as a common one. The authors acknowledge that the occurrence of child marriage is linked to lack of financial support and guidance, as well as proper access to security. The report also finds that early child marriage is linked to limited reproductive rights, including inability to choose the age of pregnancy or the use of contraceptives, and high risk of HIV.	Motives for child marriage: financial constraints and traditions.
Cherri et al. [27]	To determine the sexual and reproductive health needs of Syrian refugees in Lebanon (aged 15–49).	Focus group discussion, qualitative study.	Eleven focus group discussions were conducted across four refugee centres in Lebanon.	Early marriage was a recent trend that most women acknowledged. This was largely due to financial challenges and the rising presence of uncertainty including regarding access to housing and basic facilities. Married women also highlight issues linked to costs as a main barrier in contraceptive use, with the need to increase awareness of subsidised sexual and reproductive health services.	Impact of child marriage on child: lack of agency regarding reproductive rights; lack of access and lack of knowledge.
El-Mowafi et al. [26]	To explore Syrian child brides' knowledge of and attitudes towards experiences with contraceptive use by examining refugee camps in Jordan.	Qualitative research using focus group discussion.	The researcher conducted six focus group discussions with women and girls who married before the age of 18.	The findings show that all early marriages are through family members and most women and girls become pregnant repeatedly, with a lack of support regarding their personal choice. Many experience violence (physical and sexual).	Reproductive rights-related themes: lack of agency combined with parental pressure; lack of knowledge; willingness to learn about emergency contraceptive options.
Sahbani et al. [30]	To understand the challenges faced by international humanitarian agencies and member states. The challenges to be addressed include the health issues faced by child marriage refugees in Jordan.	Qualitative research.	Narrative in-depth review.	The authors conclude that a range of factors contribute to the ability of a girl or a woman to make a choice regarding her sexual preferences or reproductive rights. Early marriage can negatively influence health, and early pregnancy due to lack of access to the right healthcare support can also create major challenges.	Reproductive rights-related themes: systematic abuse of vulnerable women regarding managing sexual preferences and choice of reproductive rights.
UN Women Report [32]	The aim of the study was to provide an assessment of child protection issues in Jordan and identify the needs of child brides in such conflict regions. The report aimed at understanding the risks that Syrian refugee families (women and girls) in Jordan and Syria face.	Cross sectional study. Survey research.	The research targeted Syrian refugee families living in host communities in Jordan to address their views. Quantitative and qualitative data were obtained through in-depth interviews with key community informants, focus group discussion with married and unmarried refugees, and a survey questionnaire.	The findings show that the rate of early marriage is high, and the key driver of such early marriage is linked to parental pressure, community-level honour and expectations, financial challenges, and lack of education and autonomy. The study also shows that child marriage refugees continue to face reproductive rights-related challenges even after marriage and have limited decision-making capabilities.	Financial distress; economic hardship for the parents; honour-linked marriage decisions; lack of alternative options; and need for physical security.
